# Role of personal aptitudes as determinants of incident morbidity, lifestyles, quality of life, use of the health services and mortality (DESVELA cohort): qualitative study protocol for a prospective cohort study in a hybrid analysis

**DOI:** 10.3389/fpubh.2023.1069957

**Published:** 2023-06-09

**Authors:** Yudy Young-Silva, Anna Berenguera, Constanza Jacques-Aviñó, Montserrat Gil-Girbau, Paula Arroyo-Uriarte, Xenia Chela-Alvarez, Joana Ripoll, Ruth Martí-Lluch, Rafel Ramos, Usue Elizondo-Alzola, Sandra Garcia-Martinez, Fátima Méndez-López, Olaya Tamayo-Morales, Mária Martínez-Andrés, Emma Motrico, Irene Gómez-Gómez, Roberto Fernández-Alvarez, Dolors Juvinyà-Canal, the DESVELA Cohort investigators

**Affiliations:** ^1^Unitat de Suport a la recerca Girona, Fundació Institut Universitari per a la recerca a l'Atenció Primària de Salut Jordi Goli Gurina (IDIAPJGol), Girona, Spain; ^2^Facultat d’Infermeria, Universitat de Girona, Girona, Spain; ^3^Fundació Institut Universitari per a la recerca a l'Atenció Primària de Salut Jordi Goli Gurina (IDIAPJGol), Barcelona, Spain; ^4^Universitat Autònoma de Barcelona, Bellaterra, Spain; ^5^Network on Chronicity, Primary Care, and Health Prevention and Promotion (RICAPPS), Spain; ^6^Health Technology Assessment in Primary Care and Mental Health (PRISMA) Research Group, Institut de Recerca Sant Joan de Déu, Esplugues de LLobregat, Spain; ^7^Parc Sanitari Sant Joan de Déu, San Boi de Llobregat, Spain; ^8^Primary Care Research Unit of Mallorca (IB-Salut), Balearic Health Service, Palma de Mallorca, Spain; ^9^Research Group in Primary Care and Promotion-Balearic Islands Community (GRAPP-caIB), Health Research Institute of the Balearic Islands (IdISBa), Palma de Mallorca, Spain; ^10^Vascular Health Research Group of Girona, Institut Universitari per a la Recerca a l'Atenció Primària Jordi Gol I Gurina (IDIAPJGol), Girona, Catalonia, Spain; ^11^Girona Biomedical Research Institute, Girona, Catalonia, Spain; ^12^Department of Medical Sciences, University of Girona, Girona, Spain; ^13^Primary Care Services, Catalan Institute of Health, Girona, Catalonia, Spain; ^14^Grupo de Investigación en Ciencias de la Diseminación e Implementación en Servicios Sanitarios, Instituto Investigación de Biocruces, Barakaldo, Spain; ^15^Aragonese Primary Care Research Group (GAIAP), Institute for Health Research Aragón (IIS Aragón), Zaragoza, Spain; ^16^Unidad de Investigación en Atención Primaria de Salamanca (APISAL) Instituto de Investigación Biomédica de Salamanca (IBSAL), Salamanca, Spain; ^17^Faculty of Nursing, Universidad de Castilla La Mancha, Albacete, Spain; ^18^Social and Health Research Center, Universidad de Castilla La Mancha, Cuenca, Spain; ^19^Department of Psychology, Universidad Loyola Andalucía, Seville, Spain; ^20^Ourense Health Area, SERGAS, Ourence, Spain; ^21^Centro de Saúde de Allariz, SERGAS, Allariz, Spain; ^22^I-Saúde Grup, South Galicia Health Research Institute, Vigo, Spain; ^23^Grup de recerca Salut i Atenció sanitària Universitat de Girona, Girona, Spain; ^24^Càtedra de Promoció de la Salut Universitat de Girona, Girona, Spain

**Keywords:** primary health care, health promotion, determinants of health, health behaviour, life style, quality of life, qualitative research, focus groups

## Abstract

**Introduction:**

Maintaining or acquiring healthier health-oriented behaviours and promoting physical and mental health amongst the Spanish population is a significant challenge for Primary Health Care. Although the role of personal aptitudes (characteristics of each individual) in influencing health behaviours is not yet clear, these factors, in conjunction with social determinants such as gender and social class, can create axes of social inequity that affect individuals’ opportunities to engage in health-oriented behaviours. Additionally, lack of access to health-related resources and opportunities can further exacerbate the issue for individuals with healthy personal aptitudes. Therefore, it is crucial to investigate the relationship between personal aptitudes and health behaviours, as well as their impact on health equity.

**Objectives:**

This paper outlines the development, design and rationale of a descriptive qualitative study that explores in a novel way the views and experiences on the relationship between personal aptitudes (activation, health literacy and personality traits) and their perception of health, health-oriented behaviours, quality of life and current health status.

**Method and analysis:**

This qualitative research is carried out from a phenomenological perspective. Participants will be between 35 and 74 years of age, will be recruited in Primary Health Care Centres throughout Spain from a more extensive study called DESVELA Cohort. Theoretical sampling will be carried out. Data will be collected through video and audio recording of 16 focus groups in total, which are planned to be held in 8 different Autonomous Communities, and finally transcribed for a triangulated thematic analysis supported by the Atlas-ti program.

**Discussion:**

We consider it essential to understand the interaction between health-related behaviours as predictors of lifestyles in the population, so this study will delve into a subset of issues related to personality traits, activation and health literacy.

**Clinical trial registration**: ClinicalTrials.gov, identifier NCT04386135.

## Introduction

Health-related behaviours are used to understanding behaviour of individuals and the effects on their health or mortality (1). Conceptual and methodological advances have led to an increasing recognition that health behaviours are multidimensional and are integrated into lifestyles (1, 2). Furthermore, they are modified throughout life and according to the context of each individual by the “determinants of health status.” These determinants include environmental, biological, behavioural, social, economic, educational, cultural factors and the use of health services, placing the individual in his or her context (3, 4).

The social determinants of health explain most of the inequities in health, i.e., the observed unfair and avoidable differences (5, 6). In 1946, World Health Organisation (WHO) included “social welfare” in its definition of health and since then, integrating the social approach into the biomedical model has been a priority (6). However, today health inequities remain a critical problem in population health worldwide. In Spain, the direction of ordinance to reduce inequalities focuses on the distribution of power, wealth and resources in society, as well as living and working conditions (6, 7).

In this article we will focus on health-related behaviours in an attempt to understand them within a social and structural framework. We are interested in learning about people’s experiences of doing what they can or what they know, depending on their circumstances and their “personal aptitudes,” and how these experiences interact with the social and economic context of the environment. As well as with the axes of social inequality such as gender, social class, age, ethnicity and territory, amongst other contextual determinants that place people in different positions of power and opportunity.

The possible relationship of health behaviours health determinants and the presence of non-communicable diseases (NCDs) (8, 9), was raised as one of the major challenges for Health Promotion, since Ottawa 1986 where personal aptitudes were recognised as tools to achieve the development of greater control over people’s own health and the environment around them. This premise is observed from a salutogenic point of view by the WHO (10), which considers that the choices available to each individual will provide strategies for their well-being and quality of life (4). It is crucial to understand how these choices are risk factors or protective factors for health, and therefore influential in disease prevention and its treatment (11, 12).

As part of this important task, it is necessary to study how these behaviours are gathered and interact with each other. Enroot from identities that usually arise from social groups, beginning at the perspective of a healthy lifestyle, thus it is estimated that health-related behaviours account for at least 40% of deaths in the United States (13, 14). For example, some inquiry studies at the relationship between attitudinal behaviours and unhealthy behaviours, like a drinker’s strong association to add other bad practices to this conducts (15). As well as a person with damaging self-care behaviours similarly to a poor diet may be more inclined not to exercise or lead to a more sedentary life, thus accumulating a number of factors that negatively affect their health (16, 17). Therefore, understanding the interaction between health behaviours is considered essential (17), and some interventions find the need to explore further the association between self-care and habits within the population (18, 19).

Some studies have reported the implications of addictive behaviours related to negative routines causing chronic conditions which could with a greater frequency in populations with low education levels and minor economic status (20, 21). Other studies explored the views of health professionals on the influence of education on the type of decisions people make regarding how education may be related to external factors that influence their quality of life (22), in their social environment, the relationship with the health professional and the patient or the way they use the health service (23).

As mentioned above, we will look at health-related behaviours that affect lifestyle habits and become risk factors for NCDs (24). A WHO report finds that amongst the NCDs, of most concern are physical inactivity, diet, sleep, alcohol and tobacco use, sedentary lifestyle, adherence to medical treatment, and health care seeking behaviours (25, 26). Insufficient physical activity and malnutrition have led to an increase in overweight, with 16% of the population in Europe currently suffering from obesity, affecting more women than men. According to the WHO Regional Office for Europe, there is a strong relationship between obesity and low socioeconomic status, especially for women with lower level of education (preschool or primary school) (27–29). In the United States, a recent study showed that obese men have a higher prevalence of depression or depression-related symptoms compared to women (30). In concordance, to the Centres for Disease Control and Prevention, in 2021, non-Hispanic black individuals (49.6%) had the highest prevalence of obesity, followed by Hispanic individuals (44.8%), non-Hispanic white individuals (42.2%) and non-Hispanic Asian individuals (17.4%). Whilst in Spain, a study with patients with type 2 diabetes found that personality the traits, such as impulsivity, are associated with weight loss (31, 32).

The WHO also listed Europe as the continent with the highest levels of total alcohol consumption *per capita* of 10–12 L of pure alcohol during 2018 (33). A study conducted in 20 European countries found a strong association with higher alcohol consumption and poor diet, occurring more frequently in people with poorer working conditions and mostly in women (34).

Stress and sedentary lifestyles also cause NCDs. In Europe, 54.3% of people are sedentary to some degree (35). Similarly, psychological conditions that have difficult diagnosis lead to a drain on health systems, as is the case in Europe today with public expenditure on mental health *per capita* at 22%, more than double that of the Americas and the Eastern Mediterranean (36). And in many cases, are not taken into account, epidemiological data affecting these phenomena, such as African Americans having lower rates of psychiatric problems, despite their relatively higher risk of poorer health outcomes (4).

In Spain, there are also worrying figures, according to the primary data from the national health system, it has a prevalence of obesity of 24% (37) the prevalence of insufficient physical activity is 36%, and the frequency of daily tobacco consumption and weekly alcohol consumption is 22% and 37%, respectively (38). However, there are no recent studies at the national level that help to understand the interaction between these health behaviours and the regional, cultural, gender, race, education or socioeconomic patterns. Therefore, it is considered essential that qualitative or hybrid studies be carried out in Spain that would help to find these answers.

In a recent study, it was found that during confinement, health-oriented behaviours differed by gender, with women being more responsible for health promotion by caring for their children and men focusing on self-care by cooking healthy meals (39). To improve health, the heterogeneity of people and their context must be considered, and traditional gender roles must be changed. In addition, other studies suggest that emotional intelligence is positively related to healthy behaviours, life satisfaction and improved health perception, especially in the ability to relate socially (40, 41). In addition, one important study has found a positive relationship between emotional intelligence and physical and mental health, as well as health-related behaviours (42), however, there is a lack of in-depth consideration of how personal aptitudes and emotional intelligence interact with the social and economic context of participants, nor do they consider in detail the axes of social inequality and other contextual determinants of health inequalities.

Personality traits that assess a person’s set of psychological and behavioural characteristics and internal organisation, which cause him or her to act differently in a given circumstance (43); personality traits such as neuroticism (tendency to experience negative emotions), hostility and harshness are related to detrimental health behaviours, physical and mental health problems that lead to stress levels that induce lower longevity (44, 45).

Activation is defined as the ability and competences to manage one’s personal condition, maintain one’s health functioning, and make appropriate choices to prevent health deterioration. It is a tool that enables individuals to maintain health-oriented behaviours and optimise quality of life, higher levels of activation are associated with better self-care capacity and better health outcomes (46–48).

Health literacy, which values individual knowledge, motivation and competences to understand and make decisions related to health promotion and maintenance (49). Furthermore, good health literacy has been associated with healthier health-oriented behaviours. On the other hand, inadequate levels of health literacy are a critical factor in the discernment of healthy habits and can hinder self-care for chronic diseases. In consequence, improving health literacy is an unavoidable challenge from a social and health point of view (50).

Therefore, the main objective of the study is to explore participants’ experiences of health behaviours in relation to their personal aptitudes (activation, personality traits) and health literacy. In addition, the specific objectives are:

- To understand the dynamics of health behaviours according to participants’ quality of life and health status.
- To explore participants’ views and experiences of health-related behaviours according to their different local, social, economic or educational contexts.

## Methods

### Study design and characteristics

This protocol has been written based on the current literature, following the Standards for Reporting Qualitative Research (51). It is intended to be an exploratory/interpretative qualitative study, the results of the study can be of great value and highly applicable in qualitative research as a means to explore and understand the meaning that individuals or groups attribute to a social or health problem (52, 53).

This research will have a descriptive phenomenological perspective (54, 55), which aims to identify human experiences of a phenomenon as described by the participants. Therefore, research is directed at producing knowledge based on paradigmatic coherence (56).

### Context

**Figure 1:**
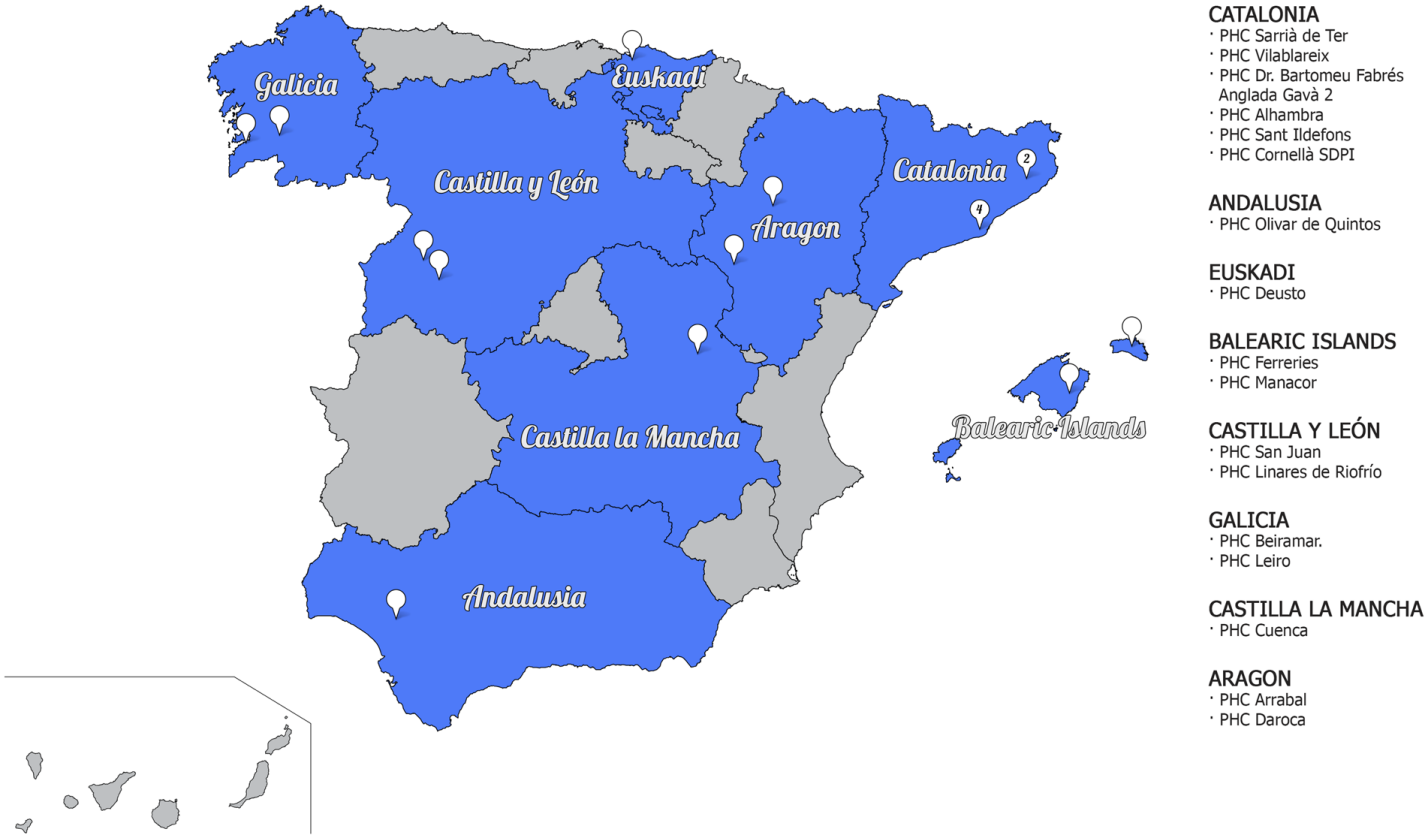
Representative map of the Autonomous Communities (AACC) and Primary Healthcare Centres (PHCs) participating in the study.

The study will be carried out within the framework of primary care and health promotion development, during the 2021–2023 period in different Primary Health Care (PHC) in Spain (see Figure 1). It is important to highlight that PHC is a level of health care where it is provided at the first contact of the patient with the health system, therefore, it is an ideal context to improve health promotion and health literacy. Thus, the PHC approach is a means to address different aspects, such as compliance with therapeutic recommendations, use of health services, follow-up of preventive measures, adoption of healthy lifestyles, amongst others.

### Participants and recruitment

**Figure 2:**
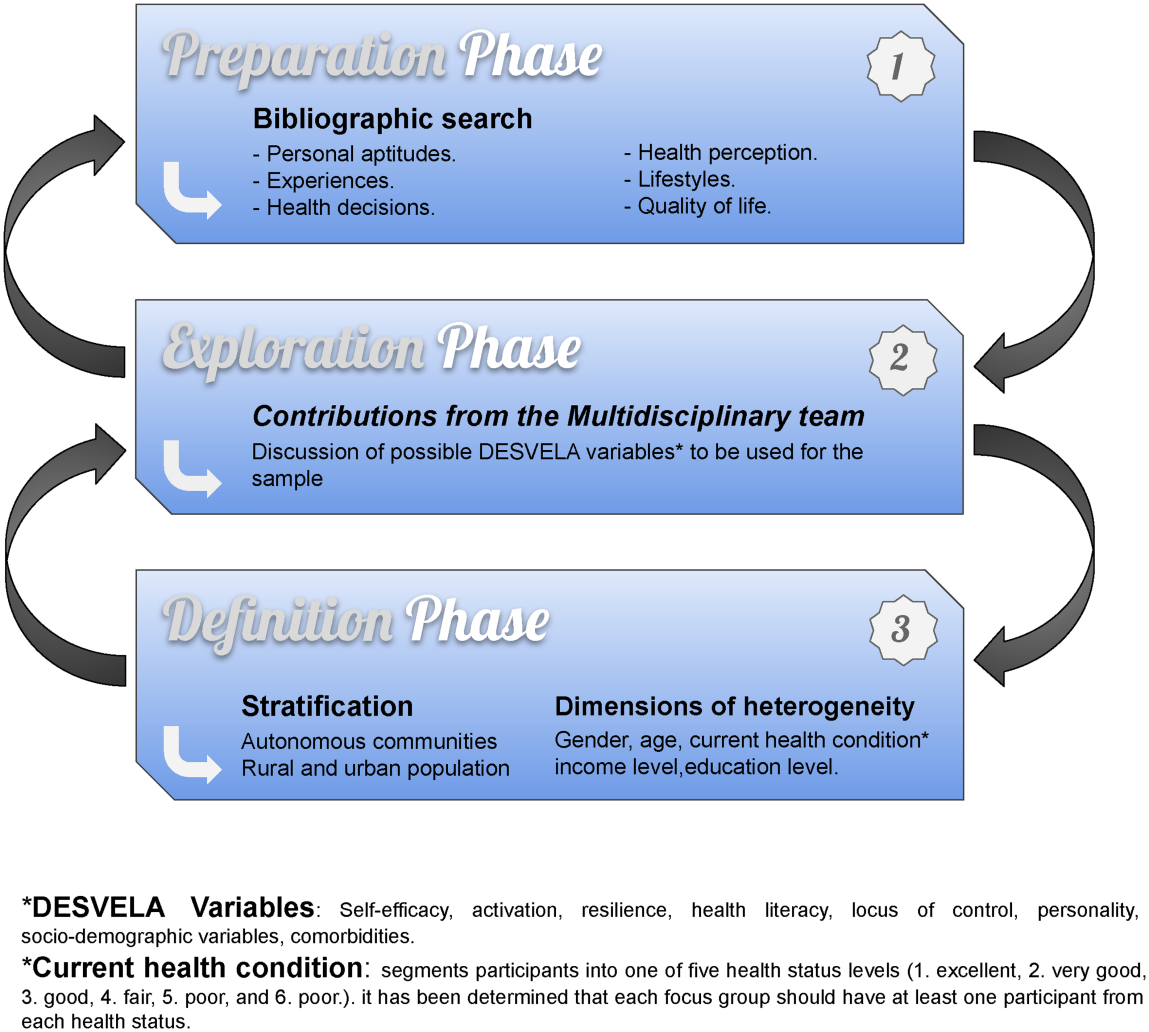
Diagram theoretical purposive sampling phase diagram.

The participants of the qualitative study will be taken from the first phase of the project as it is a mixed-methods study, i.e., they will be selected from the quantitative phase that is carried out in parallel to this qualitative study, as part of a single project called the DESVELA Cohort. Users will be recruited from PHCs in 8 Autonomous Communities (AACC) in Spain (Catalonia, Euskadi, Castilla y León, Aragón, Galicia, Balearic Islands, Castilla la Mancha and Andalusia; see Figure 1). The sample will be selected by convenience sampling based on inclusion criteria and heterogeneity variables, and will be between 35 and 74 years of age (see Figure 2).

Recruitment will be carried out by means of a telephone invitation to people who have previously expressed interest in being interviewed. All respondents will be provided with a consent form outlining the purpose and conditions for participating in the study, and will be given detailed information about their rights so that they can withdraw at any time.

### Sampling

We have created a multidisciplinary team that will support the whole process of the qualitative study, in which an initial bibliographic search was carried out to determine which of the personal aptitudes would be more suitable in order to support and expand the results of the DESVELA cohort.

The search provided us information, which was used to determine heterogeneity dimensions or factors most commonly used in other similar studies and, the most suitable dimensions for the plurality of the theoretical sampling were defined (see Figure 2).

Main dimensions for stratifying the qualitative sample:

- Autonomous communities
- Rural and urban population.

Heterogeneity Dimensions:

- Gender
- Age
- Current health condition (determined by the question “How is your general health?,” dividing the participants into 5 levels; see Figure 2)
- Income level
- Education level.

The power of the sample in a qualitative study does not depend on the size but rather on the representativeness of the discourse (57), therefore, the dimensions of stratification and heterogeneity were chosen to bring variability to the sample and reach a maximum discursive plurality (54).

The sample size will depend on the effectiveness of recruitment. Participants will be selected from the quantitative phase of the DESVELA cohort. It is estimated that each research group in the 8 AACC will recruit an average of 24 participants who will form two Focus Group Discussions (FGD) in each AACC. This will result in a total of 16 FGD, made up of 8 or 12 people, giving a maximum sample size of *n* = 192.

### Information generation techniques

This study is based on FGD, data collection technique, which involves conducting dialogue with several individuals to gather their perspectives and understand the meanings and significance of the study topics (58). The aim of FGD is to encourage interaction through sharing experiences and creating synergy between the research teams and the participants.

A person with the necessary experience will moderate the dynamics of the participants and be supported by a person who will observe the whole process and take field notes. The FGD will be conducted in varied contexts, as each AACC has different characteristics, e.g., social policies may vary according to the community, some have different local language, traditions, and food. It will take place in a free-speaking and relaxed atmosphere in an accessible location that has a space that allows for an adequate interviewing time of approximately 90 min.

Before starting the semi-structured interview questions (see Supplementary material), the FGD participants will be asked to help us describe their opinion, how good and how bad their current state of health is, by rating a scale similar to a thermometer, by rating on a thermometer-like scale (Visual Analogue Scale) (59), with a 100 for the best health you can imagine and a 0 for the worst health you can imagine.

The Focus Group Theme Script (FGTS) will be followed for the execution of the interviews (see Supplementary material). The interviews will be video and audio recorded, which will be piloted before starting the fieldwork. The FGTS is composed of 7 themes according to the results of the theoretical sampling (see Figure 2) activation, current health status, personality traits, health perception, quality of life, health habits, health literacy. The structure of the FGTS poses starter questions and the innovative implementation of 3 energiser exercises that will stimulate spontaneous participation and take advantage of group interaction to obtain better results (attached as Supplementary material).

If some participants are unable to attend the FGD and only if it is deemed necessary to achieve saturation of the discourse, some individual in-depth interviews will be conducted in which the researcher will encourage the participant to expand on the topics of discussion in detail.

### Data analysis

The Colaizzi descriptive phenomenological method will be used to analyse the data (60). The analysis will be centred on one of the AACC participating in the study, namely Catalonia, and will be a thematic and interpretative content analysis.

First, the video and audio recordings will be transcribed verbatim and anonymized. Having this information, the analyst will read the transcripts, and make some pre-analytic intuitions to identify relevant discourses within the text before dividing the text into units of meaning (familiar with the data).

The texts will be read more than once, the audios and videos will be listened to and watched as many times as necessary, and the field notes and the written exercises that were carried out as part of the FGD will be reviewed (reading-rereading), so as to obtain the codes by analogy, which will serve to classify the discourses, capturing the most outstanding ideas that respond to the narrative results obtained. The Atlas-Ti programme will be used to support the storage and organisation of all the information and data extraction (coding) and the meanings will be grouped into common themes (categorisation) (61).

Reflexivity will accompany the whole study (58). In addition, together with different researchers from different disciplines they will discuss all the data found by means of the triangulation of analysts (57), Triangulation will allow the research team to reach a consensus to interpret the phenomenon, describe the terms found and establish the relationship between the results and the objective of the study (62). Finally, a summary of the results will be returned to the participants asking them to describe their experience (validation).

## Discussion

The study will delve into the opinions and experiences of the Spanish population on the relationship between personal aptitudes and their perception of health, health-oriented behaviours and their quality of life. It will be investigated from a cross-sectional perspective (46), taking into account the age, gender, orientation, ethnicity and socioeconomic level of the study population, as well as the cultural variability provided by the different AACC involved. Our sample variability can be considered a strength; however, it can be affected by the different conditions presented in each AACC.

Currently, the leading causes of mortality and morbidity are related to health-oriented behaviours. It is investigated whether personality can influence people’s decisions on their health care and quality of life (63), it is, therefore, essential to study its influence and their relationship with NCDs and chronic diseases. Some studies assess health behaviours, but insufficient evidence has been found from a PHCs perspective on the relationship between personality, health-oriented behaviours, quality of life and health status, highlighting the need for this study.

As limitations, it should be noted that the results of this study will not be generalizable but may be transferable to other geographical contexts. On the other hand, despite the different strategies, the sample may present barriers to participation when agreeing with several people in one place at the same time. However, we expect good results thanks to the invitation to participate call and the experience of the steering group in similar studies.

Participants’ speeches can also be affected by being self-conscious about having to talk about their experiences in public, therefore, the energising exercises set out in the FGTS (see in Supplementary material) will help reduce these problems by encouraging participants to reflect by provoking instinctive reactions that will help to uncover participants’ first reactions without any influences (64). This is a strategy to make FGD more interactive, increase reflexivity, increase participants’ understanding, make the session more enjoyable and reduce social desirability bias (65).

It is hoped that the results of this study support the results of the quantitative phase of the cohort and also will help to understand health behaviours and provide sufficient and relevant information that can be applied in health care promotion campaigns, as well as contribute to the dissemination of data for health improvement through publications in scientific journals, presentations at academic meetings, workshops or local and national media.

### Strengths of this study

- This study has a multidisciplinary team that will apply reflexivity as a transversal aspect throughout the project, as well as supporting all processes of rigour and validity.
- The study will help gain a deeper understanding of the relationship between personal aptitudes and current health status amongst users of PHCs in Spain.
- The qualitative methodology of this study will complement the information of the multicentre project or DESVELA Cohort.
- Intersectionality is part of the perspective applied in the selection criteria, which gives diversity to the theoretical sample.

## Ethics statement

This project has been approved by the Clinical Research Ethics Committee from all the participant institutions: Fundació Institut Universitari per a la recerca a l’Atenció Primària de Salut Jordi Gol i Gurina (reference number 19/150-P); Comité de Ética de la Investigación con medicamentos del Área de Salud de Salamanca (reference number PI 2020 02424); Andalusian Ministry of Health, Spain (reference number: 1260-M1-21); Comité de Ética de la Investigación de medicamentos de Euskadi (CEIm-E; reference number: PI2020185); Hospital Virgen de la Luz Clinical Research Ethics Committe, Cuenca, Spain (Reference number 2019/PI2119). Research Central Commission of the Primary Care Assistance Management, Madrid, Spain (Reference Number 07/21); Comité de Ética de la Investigación de la Comunidad Autónoma de Aragón (reference number: PI20/302); Galician Ministry of health, Spain: high impact study authorization (Reference number: 2021/047). International ethical principles according to the declaration of Helsinki will be complied with. Permission will be sought before recording the interview and each participant will be assigned an identification code to protect their privacy. Collection, processing, communication, and transference of participants’ personal data will comply with the General Regulation (EU) on data protection (GDPR 2016/679) and the applicable national legislation, Organic Law 3/2018, of December 5, on the Protection of Personal Data.

## Author contributions

AB, CJ-A, DJ-C, and YY-S conceived the qualitative part of the study and participated in its design. YY-S wrote the drafts and the final version of the manuscript. AB, CJ-A, DJ-C, MG-G, PA-U, XC-A, and JR contributed to the editing of the manuscript. MG-G, PA-U, XC-A, JR, RM-L, RR, UE, SG, FM, OT-M, MM, EM, IG, and RF advised and contributed to the design of the study. All authors contributed to the article and approved the submitted version.

## DESVELA Cohort investigators

**Andalusia**—Loyola Andalucía University: Emma Motrico and Irene Gómez-Gómez and Instituto de Investigación Biomédica de Málaga: Patricia, Moreno-Peral, Sonia Conejo-Cerón, and Juan Ángel Bellón. **Aragon**—Fundación Instituto de Investigación Sanitaria Aragón: Rosa Magallón-Botaya, Fátima Méndez-López, Alejandra Aguilar-Latorre, Maria Beltrán-Ruiz, Bárbara Oliván-Blázquez, Marta Domínguez-García, María Isabel Rabanaque Hernández, and Eva María Andrés Esteban. **Castilla La Mancha**—Centro de estudios sociosanitarios: Blanca Notario Pacheco, Montserrat Solera Martínez, Lidia Lucas-de la Cruz, Miriam Garrido Miguel, María Martínez Andrés, María Eugenia Visier Alfonso, and Irene Marcilla Toribio. **Castilla y León**—Salamanca Primary Care Research Unit: José A MaderueloFernández, Leticia Sierra-Martínez, Olaya Tamayo-Morales, Miriam Daniela GarcíaCubillas, Ana B. Castro-Rivero, María D. Martín-Santos, Carmen Castaño Sánchez, and Luis García-Ortiz. **Catalunya**—Fundació Institut Universitari per a la recerca a l’Atenció Primària de Salut Jordi Gol i Gurina: Bonaventura Bolíbar, Ruth Martí-Lluch, Rafel Ramos, Yudy Young-Silva, Marc Casajuana-Closas, Anna Berenguera, Constanza Jacques-Aviñó, Lia Alves-Cabratosa, Lluís Zacarías-Pons, Anna Ponjoan, Eva Espigulé-Ribas, Francesc Ribas-Aulinas, Jordi Blanch, Èric Tornabell-Noguera, and Anna Moleras-Serra; Sant Joan de Déu Health Park: Enric Vicens-Pons, Montserrat Gil-Girbau, Mari Carmen Olmos Palenzuela, María del Carmen Gallardo González, Mª Teresa Peñarrubia-María, and Paula Arroyo-Uriarte; and Community of Madrid, Infanta Mercedes Health Centre, Madrid Health Service: Francisco Camarelles Guillem. **Euskadi**—Institute for Health Research Biocruces Bizkaia: Jose María Aiarzaguena, Álvaro Sánchez Pérez, Sandra Garcia-Martinez, Usue Elizondo Alzola, Mónica Miranda de la Maza, Ainhoa Abrisketa Ullibarri, and Mikel Rueda-Etxebarria. **Galicia**—Galicia Sur Research Institute: Mª José Fernández Domínguez, Sabela Couso Viana, Roberto Fernández Alvarez, Ana Claveria Fontan, Ana Isabel Castaño Carou, Clara González Formoso, María Victoria Martín Miguel, and Clara Guede Fernández. **Balearic Islands**—Gerencia de atención primaria de Mallorca, Instituto de investigación sanitaria de las Islas Baleares: Joan Llobera Cànaves, Caterina Vicens, Maria J. Serrano-Ripoll, Laura Gallardo-Alfaro, Oana Bulilete, Christian Jean-Mairet Soler, David Medina-Bombardó, and TColl Benejam.

## Funding

This study has been funded by Instituto de Salud Carlos III (ISCIII) with competitive grants for the period 2019–2022 through the Fondo de Investigación Para la Salud (FIS), which is co-funded by European Regional Development Fund /European Social Fund “A way to make Europe”/“Investing in your future”. Project Grants codes are: P19/01285; P19/00997; P19/01140; P19/00147; P19/01076; P19/00434; P19/01459; P19/01314; P19/01264; and P19/00115. The coordinator group received a pre-doctoral training contract in health research (PFIS-FI20/00270) from the 2020 call of the Strategic Action in Health 2017–2020, co-funded by the European Union–Next Generation EU. Investigation groups were also funded through the Research Network in Preventive Activities and Health Promotion in Primary Care (redIAPP), RD16/0007/0001; RD16/0007/0002; RD16/0007/0003; RD16/0007/0004; RD16/0007/0005; RD16/0007/0006; RD16/0007/0008; RD16/0007/0009; RD16/0007/0010; and RD16/0007/0012; and through the research grants on the call for the creation of Health Outcomes-Oriented Cooperative Research Networks (RICORS) co-funded with European Union-Next Generation EU funds, allowing the creation of the Network for Research on Chronicity, Primary Care, and Health Promotion (RICAPPS) with the following references: RD21/0016/0001; RD21/0016/0003; RD21/0016/0005; RD21/0016/0009; RD21/0016/0010; RD21/0016/0012; RD21/0016/0018; RD21/0016/0022; RD21/0016/0025; and RD21/0016/0029,. Additional grants: Regional Gerencia Regional de Salud de Castilla y León (GRS 2306/B/21 and GRS 2356/B/21); Andalusia Ministry of Education and Science (PY20 RE 025). The funders had no role in the study design, writing of the report, or in the decision to submit the protocol for publication. All authors confirm that they worked independently from funders.

## Conflict of interest

The authors declare that the research was conducted in the absence of any commercial or financial relationships that could be construed as a potential conflict of interest.

## Publisher’s note

All claims expressed in this article are solely those of the authors and do not necessarily represent those of their affiliated organizations, or those of the publisher, the editors and the reviewers. Any product that may be evaluated in this article, or claim that may be made by its manufacturer, is not guaranteed or endorsed by the publisher.

## Abbreviations

PHC: Primary health cares
WHO: World Health Organisation
NCDs: Non-communicable diseases
AACC: Autonomous communities
FGD: Focus group discussions
FGTS: The Focus Group Theme Script
