# Supplementary material for: Role of personal aptitudes as determinants of incident morbidity, lifestyles, quality of life, use of the health services and mortality (DESVELA cohort): qualitative study protocol for a prospective cohort study in a hybrid analysis
Source: Front Public Health. 2023 Jun 9;11:1069957. doi: 10.3389/fpubh.2023.1069957 (PMC10289184; doi:10.3389/fpubh.2023.1069957)
Supplement: Supplementary file 1 [file Data_Sheet_1.pdf]

DESVELA FOCUS GROUP TOPIC SCRIPT.

The focus group will address the main issues under study: Activation, current health status, personality traits, health perception, quality of life, health habits, health literacy.

1. ***Greeting, thanking and introduction of the observer interviewer***
   - Introduction of the moderator and observer: who they are, where they work, briefly clarify their role in the interview.

We are the Research Network on Preventive and Health Promotion Activities (redIAPP) which includes several research groups that want to generate knowledge to improve health control in health promotion and disease prevention in primary care.

- Welcome, thank you, your participation is really important for us.

Welcome to our session. Thank you for taking the time to join us. Your presence in this group is very important, as your opinions and contributions will help us better understand how personal skills influence people's health and quality of life.

2. ***General information on the topic to be discussed and the purpose of the session***

- General explanation of the study

This study will help obtain relevant information to explore and understand whether personal skills related to behaviours influence lifestyles, physical health, mental health, quality of life, and current health status.

We have asked you to participate in this interview because we want to hear your opinions, perceptions and experiences on the topics discussed in the interview. We are in discussions like this with various groups in 9 autonomous communities. You are invited because you were involved in the initial multi-centre project of this study, so you will already be familiar with the subject matter.

- Objectives of the focus group

This exercise aims to capture the social reality related to personal skills and people's health from the debate or discussion in this group, so you are here to talk about your personal experiences concerning the decisions you make about your health and how your personal skills influence this.

- Use of the results

We appreciate your participation in the study and this group session. Your experiences and insights are invaluable in discovering how personal skills are related to lifestyle outcomes, quality of life or the occurrence of other diseases.

The information you share during the interviews will be used confidentially to disseminate your results to different audiences (participants, research reports, congresses, conferences and publications in scientific journals).

3. Ethical and confidentiality issues:

- request informed consent and permission to record

You have probably noticed the microphone and a camera. To analyse all the content of the interview, it is essential to record the session, so we ask for your permission to record video and audio because we do not want to miss any of your comments. People often say very useful things in these discussions, and we cannot write fast enough to understand them all. The entire research team undertakes to carry out the data collection process, the analysis and the elaboration of the results confidentially and anonymously.

4. Explanation of the group interview dynamics

We raise the topics, and you can contribute whenever you want, but speaking one at a time so that we can understand the recording, the aim is to have a pleasant and relaxed conversation and for all of you to participate.

There are no wrong answers, just different points of view, all opinions are equally valuable, and everyone must express their point of view. You are free to share your views, even if they differ from what others have said. Please note that we are as interested in negative comments as we are in positive ones, and sometimes the negative comments are the most helpful.

We will use a code to identify ourselves at all times and will not use any names in our reports. You can be assured of total confidentiality.

Before we go on, do you have any questions, do you have any doubts, do you agree to participate?

5. Introduction of the participants

Well, let us get started. Let us find out little more about each other by going around the table. You can say who you are (using the code) and where you live.

6. Beginning of the interview questions and energiser exercises.

- Discuss in-depth with the help of the topic script exploring your starting questions.

Interview topics	Question to assist the moderation
Current health status - perception of health	1. What is your current health situation?<br>I feel good, or I feel bad because ...
Activation	2. Everyone needs to improve some healthy habits. What do you think you could do to improve your current state of health?<br>3. Which of your health habits or behaviours do you consider to be influenced? And you can give an example (media, society, people close to us)
health literacy	4. What difficulties do you encounter in your daily life when looking for information about your health care?<br>5. When we try to include healthy habits in our lives (eating healthier, exercising, getting enough sleep, maintaining proper mental health), which media person do we consult first?

*The blocks on the left correspond to the thematic areas to be covered, and the blocks on the right are reminders for the interviewers.

6.1 Energising exercises

These three exercises will be carried out to make the focus groups more interactive, increase reflexivity, increase participants' understanding, make it more enjoyable and reduce social desirability bias.

1. **Personality Traits - Anonymity Writing Exercise**

Participants will be asked to write down their answers on a piece of paper to give them more privacy and confidence to talk about it in the following way:

- Think of three or more personal skills that best describe you as a person and write them down on the piece of paper.

Example: I am courageous, I am a dynamic person, I am creative, I have initiative, I am self-confident, I am kind, I am patient, I am tenacious.

- Do you think these personality traits, which define you as a person, have anything to do with or influence your current health?

Example:

Because of my reserved nature, I prefer not to go to the doctor if I am ill.

My self-confidence leads me to self-prescribe if I am sick.

I feel that to be more dynamically sociable, I should consume more alcohol and foods that I have not tried on my initiative.

Write it down on paper

Quality of life - health habits

2. **Associative imagery exercise (immediate reaction)**

Participants will be shown a series of images of self-care practices and their consequences (good and bad actions) and asked to give their immediate reaction to the image.

The moderator and observer will probe for feedback, and their responses will be used to facilitate further discussion within the group. By encouraging an immediate reaction, the aim will be to capture participants' first emotions and responses to the images, decrease the social desirability bias in their responses and uncover hidden behavioural factors.

3. **Image classification (focus on what is important)**

Participants were shown pictures representing basic concepts that would help understand the level of individual quality of life, balanced and unbalanced food, smoking, alcohol consumption, sleep, sport, leisure, and others.

They will be asked to rank the images from most to least influential on health care practices. If two or more have the same influence, they may be placed side by side. After ranking the images, participants will be asked about the differences in ranking for specific elements of care,

such as having get-up-and-go, doing daily household chores, childcare, or preventing non-communicable diseases and chronic diseases. This exercise will aim to focus participants on the task and increase reflexivity.

1. **Completion of the interview**

Summary of the interview

- The moderator will make a brief summary of the participants' contributions.

Closing question

with everything that has been discussed here, how do you think your personal skills might influence your health and in what way?

Finally, any other comments you would like to add?

- Acknowledgements and reminder of the usefulness of the results, and ethical aspects.
